# Imaging for central nervous system (CNS) interstitial fluidopathy: disorders with impaired interstitial fluid dynamics

**DOI:** 10.1007/s11604-020-01017-0

**Published:** 2020-07-11

**Authors:** Toshiaki Taoka, Shinji Naganawa

**Affiliations:** 1 Department of Innovative Biomedical Visualization (iBMV), Nagoya University Graduate School of Medicine, 65 Tsurumai-cho, Showa-ku, Nagoya, Aichi 466-8550 Japan; 2 Department of Radiology, Nagoya University Graduate School of Medicine, Nagoya, Japan

**Keywords:** Interstitial fluidopathy, Interstitial fluid dynamics, Cerebrospinal fluid, Pathophysiology, Glymphatic system

## Abstract

After the introduction of the glymphatic system hypothesis, an increasing number of studies on cerebrospinal fluid and interstitial fluid dynamics within the brain have been investigated and reported. A series of diseases are known which develop due to abnormality of the glymphatic system including Alzheimer’s disease, traumatic brain injury, stroke, or other disorders. These diseases or disorders share the characteristics of the glymphatic system dysfunction or other mechanisms related to the interstitial fluid dynamics. In this review article, we propose “Central Nervous System (CNS) Interstitial Fluidopathy” as a new concept encompassing diseases whose pathologies are majorly associated with abnormal interstitial fluid dynamics. Categorizing these diseases or disorders as “CNS interstitial fluidopathies,” will promote the understanding of their mechanisms and the development of potential imaging methods for the evaluation of the disease as well as clinical methods for disease treatment or prevention. In other words, having a viewpoint of the dynamics of interstitial fluid appears relevant for understanding CNS diseases or disorders, and it would be possible to develop novel common treatment methods or medications for “CNS interstitial fluidopathies.”

## Introduction

### Proposing “Central nervous system interstitial fluidopathy”

In 2012, Iliff and Nedergaard et al. hypothesized that the cerebrospinal fluid (CSF) and interstitial fluid (ISF) in the perivascular or interstitial spaces within the brain constitute a mass transport system or pathway corresponding to the lymphatic system [1]. This system was named as the “glymphatic system,” that was coined by combining “g” for glia with “lymphatic” system. According to the glymphatic system hypothesis, the perivascular space functions as a conduit for the CSF to flow into the brain parenchyma. The driving force for this conduit is hypothesized as the arterial pulse. CSF within the perivascular space around the arteries enters the interstitial space via the water channels controlled by aquaporin-4 (AQP4), which is distributed in the astrocyte foot processes that make up the outer wall of the perivascular space. The CSF in the interstitium flushes away waste proteins in the tissue. Thereafter, the CSF, which has been washed away between cells, flows into the perivascular space around the vein and is discarded to outside the brain.

**Fig. 1:**
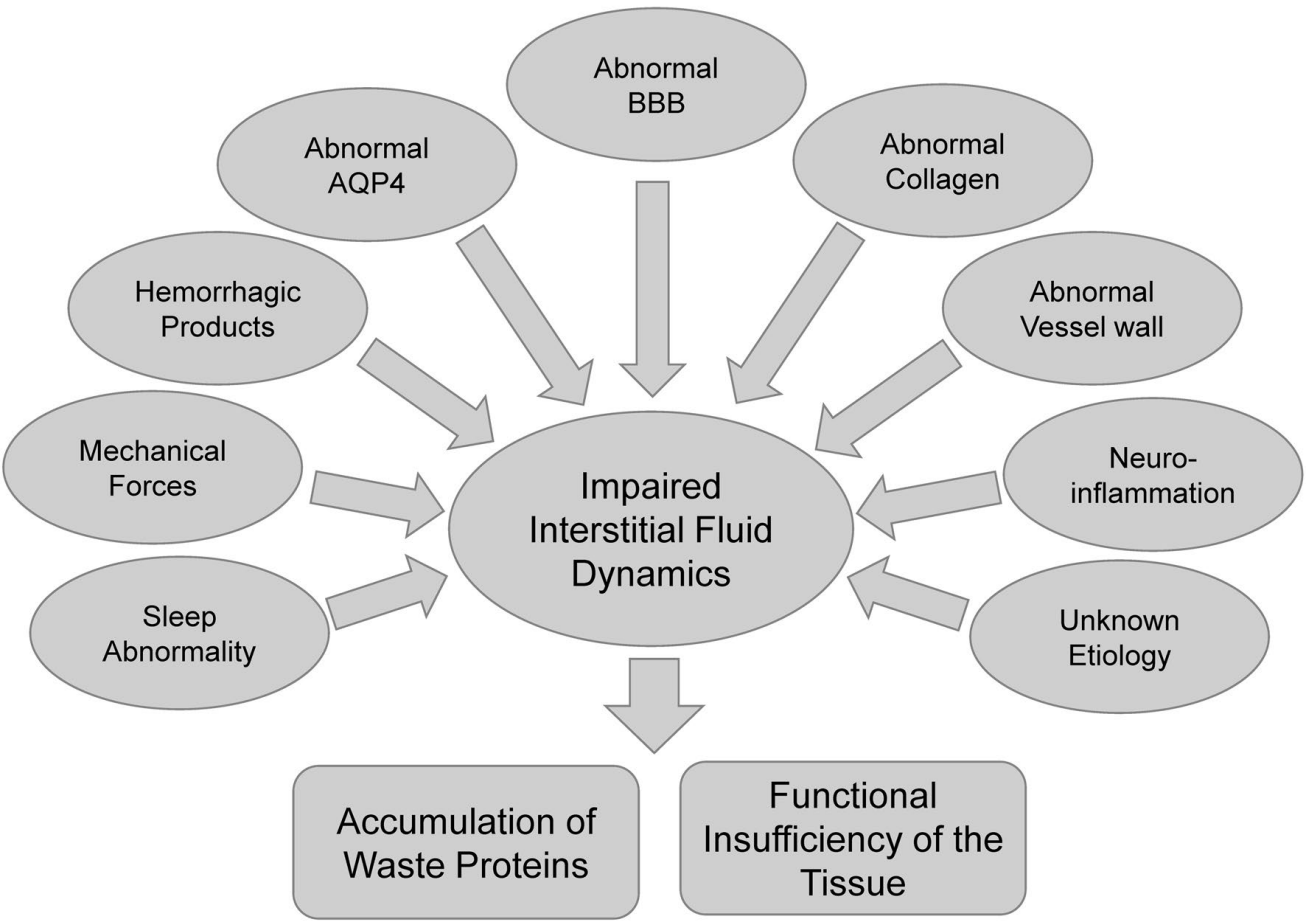
Overview of “CNS Interstitial Fluidopathy”. Different etiology including mechanical force, distribution of hemorrhagic products, and other abnormality results in impaired interstitial dynamics

Following the introduction of this glymphatic system hypothesis, an increasing number of studies were published attempting to describe the interstitial fluid dynamics within the brain. The interstitial space in the brain is regarded as a common space, that not only acts as a supportive structure, but also functions as a space for mass transport, immune function, and intercellular signal transmission. In order to describe the fluid within the brain including the interstitial space, the term “neurofluids” was used by Toro et al. as the project title of a series of studies simulating the entire fluid dynamics of the central nervous system (CNS) with a mathematical model. “Neurofluids” is defined as a collective term for the fluids in which the CNS is immersed, including the blood, CSF, and ISF [2, 3]. This term can be helpful for understanding the ISF/CSF dynamics. Several diseases of the CNS that share the dysfunction of the glymphatic system or other mechanisms related to the interstitial space or dynamics of “neurofluids.” are known. In these diseases, mechanisms related to the interstitial mass transportation or fluid dynamics will increase information and understanding of the disease process and will be helpful in its treatment or prevention in the future. Combining these discussions, we propose “CNS Interstitial Fluidopathy” as a new concept that signifies diseases in which abnormal interstitial fluid dynamics has major association with their pathology (Fig. 1). In this review article, we describe such diseases with impaired fluid dynamics of the CNS interstitial space as “CNS Interstitial Fluidopathy,” and review them from the pathophysiology and neuroimaging perspective.

## Interstitial fluid dynamics of normal CNS and imaging

Since the introduction of the glymphatic system, the perivascular and interstitial spaces have been recognized as an important stage for ISF/CSF dynamics. While a number of trials by imaging, mainly by magnetic resonance imaging (MRI), have been made to visualize or evaluate the glymphatic function or ISF/CSF dynamics.

### Perivascular space and interstitial space in CNS

The perivascular space is a space surrounding the arterioles and venules after penetrating from the subarachnoid space into the brain tissue. The arteries and veins that run in the subarachnoid space have a pial sheath that closely surrounds them. The intraparenchymal arterioles continue to have an inner pial sheath that closely surrounds the adventitia. The structure of the perivascular space is built on the endothelial, pial, and glial cell layers, and each of them is delineated by the basement membranes [4]. The outer wall of the perivascular space is formed by the glia limitans which is the glial membrane covering the brain parenchyma. At the capillary level, the perivascular space disappears as the basement membrane of the glia fuses with the outer vascular membrane.

The interstitial space in the brain occupies approximately 15–20% of the total brain volume, and it contains the ISF and extracellular matrix [5]. The ISF in the brain works as the medium for nutrient supply, waste removal, and intercellular communication [5]. The extracellular matrix is a highly hydrated net-like structure mainly composed of long-chain molecules, and it surrounds and attaches to the cell membrane [6]. The complex of capillary and astrocyte of the blood brain barrier (BBB) has been regarded as a structure producing the brain ISF, and the ISF secreted at the BBB is coupled with shifts of extracellular fluid between the brain and CSF [7]. The BBB is mainly composed of brain microvascular endothelial cells, pericytes, astrocytes, and basement membrane, and regulates the transport of molecules to protect the brain microenvironment. The basement membrane is a layer of extracellular matrix found predominantly underneath the endothelial and epithelial cells, and the interface between the epithelial cells and the underlying connective tissue matrix. The basement membrane consists of extracellular matrix proteins including collagen IV, proteoglycans, and glycoproteins such as laminin. The CSF enters the brain along the basement membrane between the pia mater and glia limitans and enters the brain parenchyma and ISF by an AQP4-dependent mechanism [8].

### Evaluation of interstitial fluid dynamics by MRI

On MRI, the perivascular spaces are seen as clusters of variably sized cystic areas, exhibiting CSF signal across all sequences. The normal perivascular spaces are generally smaller than 2 mm. Often, they occur along the path of the penetrating arteries. The perivascular spaces should not be enhanced, display abnormal restricted diffusion, or exhibit any T2-weighted image (T2WI) or FLAIR signal abnormalities. Perivascular spaces are characteristically divided into three subtypes based on the location. The first type is frequently seen on MRI and appears along the lenticulostriate arteries entering the basal ganglia, the second type can be found along the path of the perforating medullary arteries as they enter the cortical gray matter over the high convexities, and the third type appears in the midbrain at the pontomesencephalic junction [9–11].

Several MR methods are available for the evaluation of the permeability of the BBB. Dynamic contrast-enhanced MRI (DCE-MRI) is one such method. Although DCE-MRI has some technical challenges, this method has been utilized for evaluating the changes in BBB leakage [12]. One of the DCE studies has shown that the global BBB leakage in patients with early Alzheimer’s disease (AD) to be associated with cognitive decline [13]. Leakage of gadolinium-based contrast agents (GBCA) into the brain parenchyma of patients with vascular cognitive impairment has also been demonstrated by DCE-MRI studies. The disruption of the BBB in chronic vascular disease has been linked with hypoxia induced inflammation [12].

Intrathecal administration of GBCA is not approved in clinical practice. However, several published researches involving intrathecal administrations of GBCA in human patients have been performed through careful procedure, appropriate dose decision, and approval of their institutional board certification [14, 15]. One of the reports showed that GBCA was distributed in the brain following 1 and 4.5 h of intrathecal administration, which enabled visualization of the glymphatic perfusion or ISF/CSF dynamics [14]. As most of the current study reports on intrathecal GBCA are for idiopathic normal pressure hydrocephalus (iNPH), this method will be discussed again in the below chapter about “Idiopathic normal pressure hydrocephalus”.

Recently, the transport of intravenously injected GBCA into the CSF or perivascular space has been reported [16, 17]. At approximately 4 h after intravenous injection of GBCA, transport of GBCA into the perivascular space at the base of the brain can be observed on heavily T2-weighted fluid-attenuated inversion recovery images [18–24]. On delayed imaging after injection, intravenously administered GBCAs demonstrate leakage from the cortical veins, which increase with age [25, 26]. Additionally, the space between the pial sheath surrounding the cortical veins and venous wall was shown as enhanced, which appears as connected to the meningeal lymphatics that run along the superior sagittal sinus [27].

There are several imaging techniques using MRI for evaluation of ISF/CSF dynamics. Diffusion MRI includes various specialized methods for evaluating water dynamics within the cranium [28, 29]. Several studies have suggested the potential of diffusion MRI technique to investigate the glymphatic system [17, 30–33]. Arterial spin labelling (ASL) method has also been tried for evaluating the glymphatic function or ISF dynamics. A multi-echo time (TE) ASL technique to assess water transport at the BBB in a mouse brain showed a 31% increase in the exchange time in AQP4-deficient mouse, which suggests water permeability to be a key determinant of waste clearance from the brain [34]. There are trials to evaluate the ISF/CSF dynamics by chemical aspect. Lymphatic chemical- exchange- saturation- transfer imaging (Lym-CEST) is one of solution to distinguish lymph fluid from blood and CSF. The special CEST effect in lymph was observed at about 1.0 ppm from water in Z-spectrum, and in the unilateral deep cervical lymph node ligation models group, the intensity of the CEST signal was significantly higher in the ipsilateral hippocampus [35]. In an experiment to visualize the extracellular water as distinct T2 values, short T2 components reported to exist along the white matter, in the choroid plexus. These can be considered as distribution of macromolecules (waste materials) in the brain. Thus, it seems to be possible to obtain some insight into pathways for the transport of large molecules in the brain [36].

## Disorders categorized in CNS interstitial fluidopathies

In 2012, Nakashima et al. discussed that dysfunction of the ISF is associated with hydrocephalus, glaucoma and Meniere’s disease, and pointed out a common background for these diseases, despite their anatomical, physiological and clinical relationships [37]. In parallel, following the proposal of the glymphatic system hypothesis by Ilif et al. in 2012 [1], a series of studies reported on diseases which developed because of the glymphatic system abnormality. These diseases share the characteristics of the dysfunction of the glymphatic system or other mechanisms related to the dynamics of the ISF. Categorizing these diseases as “CNS interstitial fluidopathies,” promotes the understanding of the disease mechanisms and development of potential methods for disease treatment or prevention. In the following part of this review, diseases categorized as “CNS interstitial fluidopathies” will be discussed.

### Sleep disorders and ISF dynamics

One of the reasons why the glymphatic system theory has involved scientific and clinical attention is because of its correlation with sleep. Under physiological conditions, the activity of the glymphatic system is associated with sleep. Drainage by the glymphatic system is suppressed during awakening and is markedly increased during sleep, which is partially because of autonomic system. This has been reported as due to the decrease in the volume of the glial cells during sleep, and because the interstitial space expands more during sleep, facilitating tissue mass transport [38]. The volume ratio of the interstitial space in tissues is considered as 13–15% when awake, whereas 22–24% during sleep [39]. During awakening, the noradrenaline activity in the brain tissue is higher than that during sleep. Noradrenaline has been shown to increase astrocyte volume, that is, reduces interstitial volume [40].

In addition, studies have been conducted on the relationship between the development of dementia in humans, sleep, and the glymphatic system. CSF amyloid β (Aβ) levels are predictors of tissue Aβ deposition, but sleep disorders have been associated with fluctuations in CSF Aβ levels [41]. Even overnight vigilance has been reported to impair amyloid β42 reduction in physiological CSF in the morning and can be a risk of developing AD [42]. In an imaging study in a younger population, overnight changes in diffusivity were reported as a proxy marker for clearance in the glymphatic system. The mean diffusivity increased overnight in multiple brain regions, consistent with the hypothesized expansion of the extracellular space during sleep. This overnight increase in diffusivity in the brain was positively correlated with the percentage of time spent in rapid eye movement sleep [43]. These reports suggest that disorder or abnormality in sleep also affects the ISF dynamics within the brain.

### Alzheimer’s disease/Parkinson’s disease as CNS interstitial fluidopathies

AD is categorized as an amyloidopathy as well as tauopathy [44]. However, several animal experiments have indicated a profile of impaired ISF dynamics in AD [1, 45, 46]. The initial paper proposing the concept of glymphatic system presented an assessment of Aβ excretion in healthy and AQP4-knockout mice. Fluorescent-tagged Aβ, was transported along the perivascular route, and deletion of the Aqp4 gene suppressed the clearance of soluble Aβ, suggesting that the glymphatic pathway may be involved in the removal of Aβ from the CNS [1]. An experimental study indicated that ligation of deep cervical lymph nodes exacerbated AD-like phenotypes of AD model mice, demonstrating a more severe brain Aβ accumulation, neuroinflammation, synaptic protein loss, impaired polarization of AQP4, and deficits in cognitive and exploratory behaviors. These results suggested that malfunction of the brain lymphatic clearance as one of the deteriorating factors in AD progression [45].

Limited number of human studies indicating the alteration of ISF dynamics in AD has been presented. One of the studies utilized 11C-Pittsburgh compound B (PiB) PET, known for amyloid imaging as a tool for CSF clearance detection. The 11C-PiB signal in the lateral ventricles was measured for compartmental modeling, and significant group differences were observed across AD, mild cognitive impairment (MCI), and healthy controls. This result indicated the association of CSF clearance deficits in AD with Aβ deposits [47]. Another study utilized diffusion imaging to detect ISF dynamics in AD. The diffusivity along the perivascular spaces as well as projection fibers and association fibers was evaluated separately to acquire an index for diffusivity along the perivascular space (ALPS-index) and then correlated with the mini-mental state examinations (MMSE) score in AD and MCI cases. The positive correlation between diffusivity along perivascular spaces shown as ALPS-index and the MMSE score was significant, indicating lower water diffusivity along the perivascular space with respect to AD severity [48]. Therefore, AD may be regarded as one of the “CNS interstitial fluidopathies,” while with a profile of amyloidopathy or tauopathy.

Glymphatic dysfunction is suggested to be implicated in the development of Parkinson’s disease (PD), which has a profile of α-synucleinopathy, wherein aggregated alpha-synuclein deposits cause neuropathology, and neuroimaging studies have contributed to our understanding of the pathophysiology and diagnosis of PD [49–52]. In an experimental study of PD model mouse which overexpresses mutated human α-synuclein, meningeal lymphatic drainage was blocked by ligating the deep cervical lymph nodes. Glymphatic influx of CSF tracer was reduced in these model mice, causing severe accumulation of α-synuclein, glial activation, inflammation, dopaminergic neuronal loss, and motor deficits [53]. Another study indicated AQP4 deficiency as a key factor in intensifying the sensitivity of dopaminergic neurons in PD mouse models [54]. In addition to protein accumulation, the effect of dopaminergic deterioration may be crucial in the disruption of sleep and ISF dynamics in PD [55, 56]. These findings suggests that PD also has characteristics as a CNS interstitial fluidopathy.

The profiling of AD or PD as a CSF interstitial fluidopathy suggests a new approach for the treatment or prevention for these neurodegenerative diseases. Currently, a limited number of studies has been reported on the enhancement of glymphatic function. One of the reports demonstrated that n-3 polyunsaturated fatty acids (PUFAs) promote interstitial Aβ clearance from the brain. An imaging study on clarified brain tissues demonstrated that n-3 PUFAs inhibit the activation of astrocytes and protect the AQP4 polarization [57]. The cilostazol for prevention of Conversion from MCI to Dementia trial evaluating the efficacy of cilostazol in patients with MCI is ongoing in Japan [58]. Cilostazol is known to suppress platelet aggregation, protect vascular endothelia, dilate vessels, and increase cerebral blood flow. Therefore, promotion of the major vascular-mediated Aβ elimination systems, including capillary transcytosis, the glymphatic system, and intramural periarterial drainage route [59]. Although further study is required before establishing, trials for treatment or prevention of neurodegenerative diseases are claiming the improvement of ISF dynamics.

### Traumatic brain injury as a CNS interstitial fluidopathy

**Fig. 2:**
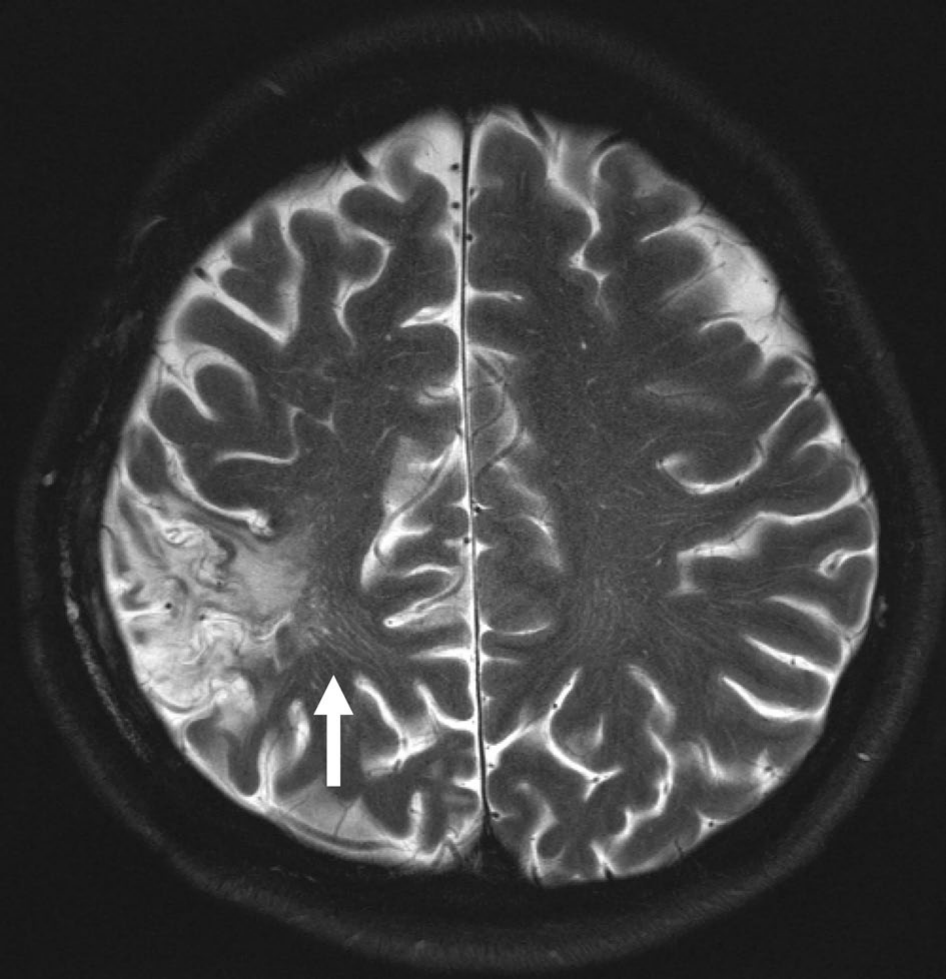
Traumatic brain damage. A 5th decade female with a history of traumatic subdural hemorrhage who underwent surgical treatment, following which she gradually developed gait disturbance. T2-WI shows dilated perivascular space especially in the region adjacent to the old traumatic brain contusion (arrow)

Several known disorders are known in which abnormal ISF dynamics is caused due to mechanical force. Iliff et al. examined a model of moderate to severe trauma in mice. They demonstrated that the migration of tracer injected into the perivascular cortex of mice after injury was significantly reduced compared with the ipsilateral region to trauma suggesting reduced activity of the glymphatic system, and this finding was continued 28 days after the injury [60]. In chronic traumatic encephalopathy, a progressive encephalopathy caused by repetitive damage to the brain, an abnormal accumulation of Aβ and TDP-43 is observed, and in recent years, tauopathy has been indicated [61]. In case of craniectomy, alteration due to a mechanical force can cause impaired ISF dynamics. Craniectomy itself, without any other insult, has been reported to alter brain function due to reduced arterial pulsatility and decreased ISF flow in an animal experiment. After unilateral craniectomy, penetrating arterial pulsatility decreased significantly causing immediate and chronic impairment of the CSF influx in the ipsilateral and contralateral brain parenchymas. This craniectomy-related dysfunction of ISF dynamics was associated with an astrocytic and microglial inflammatory response, as well as with the development of motor and cognitive deficits [62]. A Another animal experiment by using contrast-enhanced MRI indicated that repetitive mild TBI caused an increased fluid influx to the brain tissue but reduced efflux throughout the limbic structures and olfactory bulb, but neither the influx nor efflux was altered in the cortical structures [63]. The observation of MRI in human cases of mild TBI revealed that an increased number of enlarged high-convexity perivascular space was detected on T2-weighted images compared to the controls (Fig. 2). This finding might be a marker of potential inflammatory changes associated with mild TBI, especially among the young patients. The enlarged high-convexity perivascular space probably reflects early and permanent brain changes, which might reflect the accumulation of inflammatory cells and/or changes of vascular permeability [64]. These findings suggests that mechanical forces to the brain including traumatic brain injury also affects the ISF dynamics in the brain, and have the aspect of CNS interstitial fluidopathy.

### Stroke as a CNS interstitial fluidopathy

The first animal study to evaluate the glymphatic function on MRI was performed using intrathecal injection of GBCA and observing the reduced brain clearance rate of GBCA [65]. The experiment was focused on the function of the glymphatic system or ISF dynamics in mouse models of stroke, including those for subarachnoid hemorrhage (SAH), intracerebral hemorrhage, carotid ligature, and embolic ischemic stroke. It was found that the ISF dynamics was severely impaired after SAH and in the acute phase of ischemic stroke but was not altered after carotid ligature or intracerebral hemorrhage. Especially, the ISF dynamics is severely impaired after SAH, and the blockage of glymphatic drainage has been shown to worsen cerebral ischemia and edema after acute SAH [65]. The relevance of the report was in that it provided insights for the treatment of these conditions. The intra-ventricular injection of the tissue-type plasminogen activator improves the glymphatic drainage, and spontaneous arterial recanalization was associated with the restoration of the glymphatic drainage after embolic ischemic stroke. Recently, a report on experimental SAH in mice revealed deteriorated ISF dynamics. The experience showed that SAH mice displayed decreases in fluorescent tracer drainage to the deep cervical lymph nodes and influx into the brain parenchyma after tracer injection into the cisterna magna. The function of astrocyte AQP4 was impaired and resulted in accumulation of the Tau proteins in the brain. Additionally, pathological changes, including microvascular spasm, activation of glial cells, neuroinflammation, and neuronal apoptosis were observed in the hippocampus of SAH mice [66].

Huge number of clinical studies for stroke have been reported in human subjects [67–69]. Although a systematic study on the ISF dynamics on human stroke cases has not yet been reported, an interesting report has suggested the correlation between ISF dynamics and stroke. Computed tomography (CT) images of endovascular perforation cases during thrombectomy for acute ischemic stroke were retrospectively evaluated, and progressive absorption and wash out of iodine contrast media by the brain parenchyma, which means glymphatic clearance, were observed. Both hemorrhagic transformation and ischemic infarction were not developed in the areas revealing apparent wash out of iodine contrast media [70]. Even though there is limited evidence for the alteration in the ISF dynamics in stroke cases, considerations for the ISF dynamics will provide an insight or new insight for the treatment or prevention of stroke symptoms.

### Small vessel diseases of CNS

Cerebral small vessel diseases are a group of pathological processes affecting the small perforating arteries, arterioles, capillaries, and venules resulting in damage to the cerebral white and deep grey matter, which includes arteriosclerosis, amyloid angiopathy, and other genetic small vessel diseases. In these cerebral small vessel diseases, dilated perivascular space is often observed, and these diseases also show deposition of Aβ and other abnormal proteins in the tissues [71]. It is difficult to explain these two events in terms of a single pathophysiology such as damage to the vascular walls. However, given the context of glymphatic system or ISF/CSF dynamics impairment reveals that the accumulation of abnormal proteins in tissues and consequent dilation of the perivascular space may be mutually associated events [72].

Arteriolosclerosis is the most common small vessel alteration in aged brains. In the animal experiments using MRI with intrathecal administrated GBCA on spontaneously hypertensive rats, ventricular reflux of GBCA was observed only in the hypertensive rats, indicating that hypertensive rats have abnormal ISF/CSF dynamics or impaired glymphatic clearance [73]. Cerebral amyloid angiopathy shares Aβ pathology with AD, and an experimental study has demonstrated that CSF inflow and ISF clearance in the brain are suppressed in a mouse model of AD and in these the ISF/CSF dynamics is suppressed prior to the significant accumulation of Aβ [74]. One observational study on humans reported that the dilatation of perivascular space in the centrum semiovale, which may suggest impaired ISF drainage, significantly correlated with the existence of cerebral amyloid angiopathy even in the absence of any lobar hemorrhages [75].

**Fig. 3:**
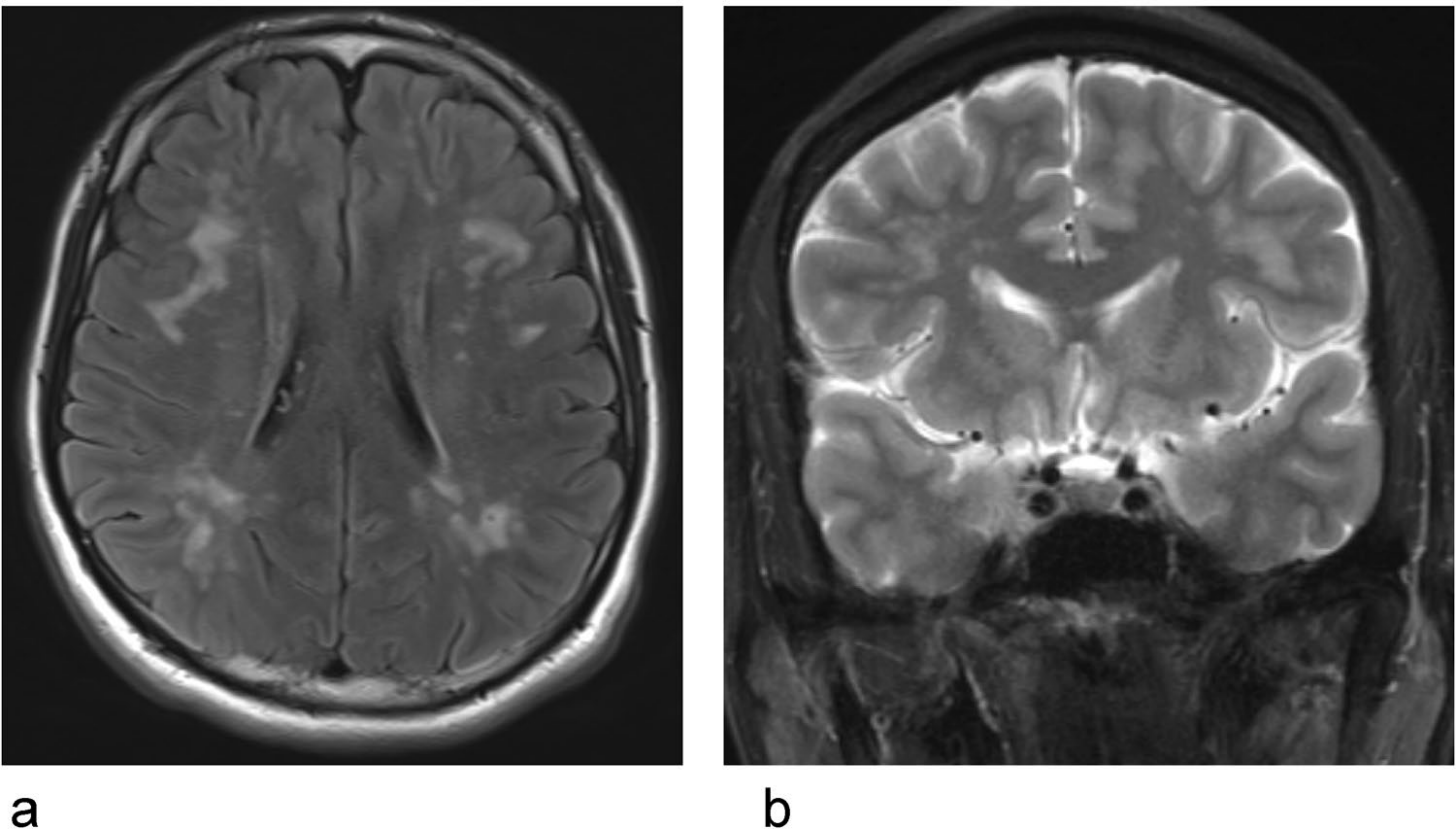
A case of small vessel disease diagnosed as CADASIL (cerebral autosomal dominant arteriopathy with subcortical infarcts and leukoencephalopathy) **a** FLAIR image, **b** T2-WI. A 5th decade male with severe migraine like symptom. Skin biopsy revealed GOM deposition and was diagnosed as CADASIL. His father had developed cerebral infarction in his 6th decade. Axial FLAIR image and coronal T2WI showing subcortical abnormal signal suggesting dilated perivascular space and surrounding gliosis

Cerebral autosomal dominant arteriopathy with subcortical infarcts and leukoencephalopathy (CADASIL) is caused by a mutation in NOTCH3 and accounts for the largest number of cases of cerebrovascular disease due to a single gene abnormality. Deposition of granular osmiophilic material (GOM), which causes smooth-muscle-cell degeneration, is found in arterioles throughout the body, but vascular degeneration is remarkable in the white matter region of the brain. Due to vascular smooth-muscle-cell degeneration, dysfunction in the ISF/CSF dynamics may occur, causing impaired excretion of GOM, which may lead to a vicious cycle of GOM deposition [72, 76]. On MRI, dilated perivascular spaces are frequent in CADASIL and mostly located in the temporal white matter and basal ganglia (Fig. 3). The dilation of perivascular spaces does not appear to be directly related to the occurrence of ischemic or hemorrhagic lesions, while its relation with age suggests either aging, progression of vascular wall alterations during the course of the disease [77]. While CADASIL causes impaired ISF dynamics mainly due to the degeneration of smooth muscle in the vascular wall, there are other hereditary small vessel diseases related to other structures of vascular, perivascular, and interstitial structures [78]. COL4A1 mutation related disorders are part of hereditary small vessel diseases and are characterized as cerebral small vessel diseases with diverse disease phenotypes that include porencephaly, stroke, glaucoma, and other angiopathies. COL4A1 is a gene encoding type IV collagen alpha-1 chain. As a cerebral small vessel disease, COL4A1 related disorders cause familial vasculopathy and may present with ischemic as well as hemorrhagic stroke, in adult life with radiological features of leukoaraiosis and microbleeds [79]. Collagen 4 is exclusively found in the basement membranes, and is a nonfibrillar collagen which is categorized into network, mesh, and sheet forming collagen [80]. Histologic evaluation of brain tissues in autopsy has revealed irregular thickening, disruption, splitting, and fragmentation of the capillary basement membrane along with the accumulation of pools of basement membrane fragments [81]. As cerebral vascular basement membranes form the pathways by which fluid passes in and out of the brain [8], the alteration of the basement membrane in COL4A1 related disorders probably causes impaired ISF/CSF dynamics.

### Glaucoma

**Fig. 4:**
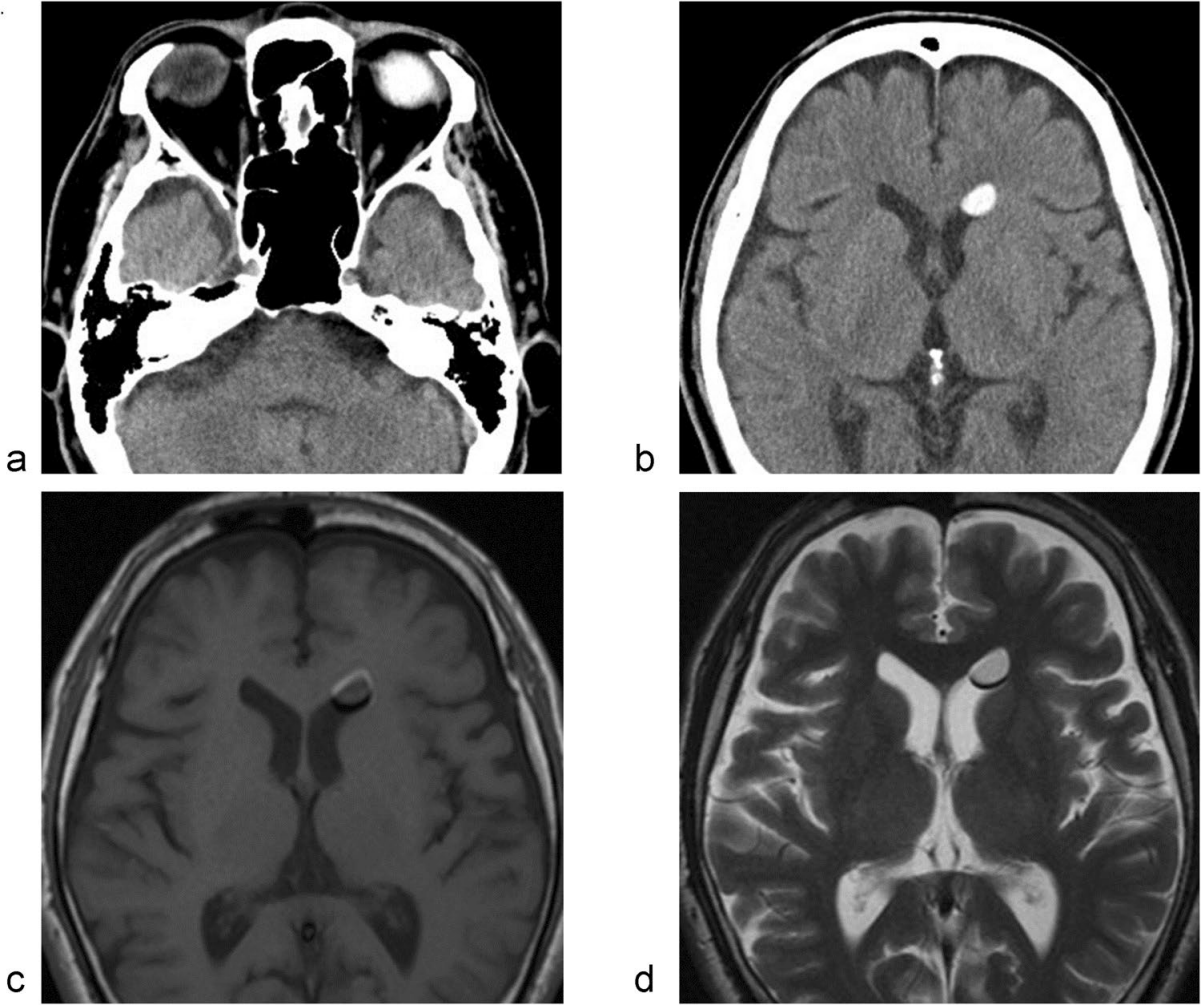
Migration of intraocular silicone oil into the brain. **a**, **b** CT, **c** T1-WI, **d** T2-WI. A 6th decade male who has silicone oil tamponade in vitreoretinal surgery for retinal detachment. After 10 years of the surgery, brain CT revealed high density material in his lateral ventricle. MRI showed chemical shift on both T1 and T2-WI, indicating silicone oil transport into the brain. The migration of the silicone oil is speculated to have taken place via the subarachnoidal space around the optic nerve

The optic nerve, a white matter tract of the CNS, is ensheathed in all three meningeal layers and surrounded by CSF with a pressure equivalent to the intracranial pressure, and the existence of a perivascular transport system in the retina and the optic nerve has been hypothesized, which might be an important factor in retinal diseases, such as age-related macular degeneration and glaucoma [82, 83]. Recently, Aβ was demonstrated as cleared from the retina and vitreous via a pathway dependent on the glial water channel AQP4, driven by the ocular-cranial pressure difference. In an experiment in rodents, intra-axonal Aβ was cleared via the perivenous space and subsequently drained into the lymphatic vessels. Interestingly, light-induced pupil constriction enhanced efflux, while atropine or raising intracranial pressure blocked efflux [84]. Additionally, in humans, communication of ISF between the eye and brain has been suggested [37]. Silicone oil has been used as an intraocular endotamponade for the repair of complex retinal detachment. Several reports on the cases of silicone oil migration into the chambers of the eye, the periorbital space, and into the cerebral ventricles are known [85–87] (Fig. 4). Based on these findings, in glaucoma, it is hypothesized that an alteration of fluid dynamics in the intraocular and intracranial spaces may result in impaired CSF entry into the subarachnoid and perivascular spaces of the optic nerve, thereby inhibiting glymphatic clearance of waste products from the retrobulbar or retrolaminar portion of the optic nerve [88]. Additionally, these mechanisms share the characteristics of dysfunction of the glymphatic system or dynamics of ISF in the retina and optic nerve in glaucoma cases, which could be categorized as an interstitial fluidopathy.

### Meniere’s disease

**Fig. 5:**
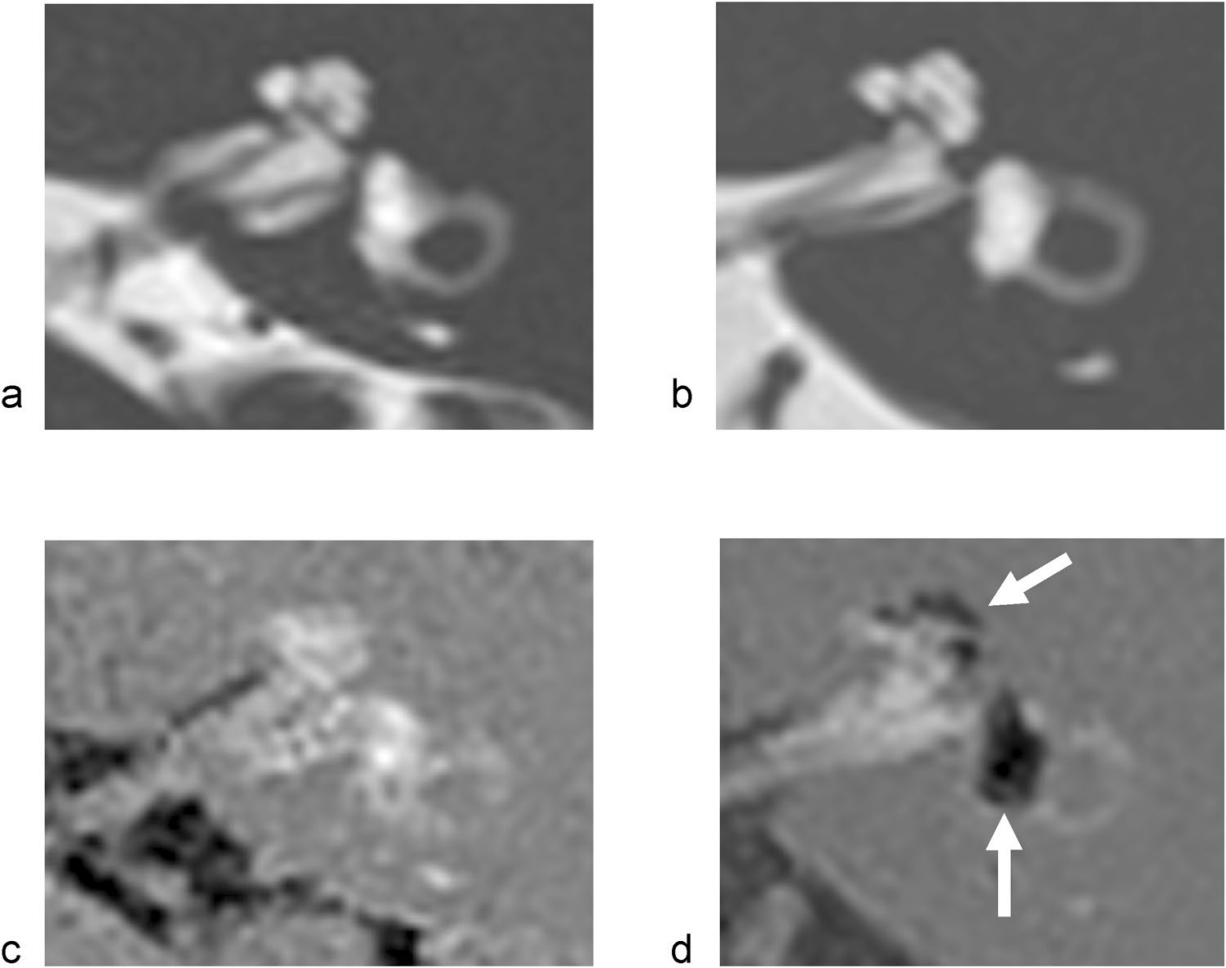
MRI of endolymphatic hydrops in Meniere's disease. **a**, **b** MR cisternography, **c**, **d** Hybrid of reversed image of positive endolymph signal and native image of positive perilymph signal (HYDROPS) image. Healthy (**a**, **c**) and a Meniere’s disease ear (**b**, **d**) are presented. MR cisternography (**a**, **b**) shows a high signal both for the inner ear labyrinthine endolymph and perilymph. On HYDROPS image obtained 4 h after intravenous administration of single-dose gadolinium-based contrast agent (GBCA) (**c**, **d**), endolymph is delineated as low signal since GBCA does not transit into endolymph. In Meniere’s disease ear, dilatation of endolymphatic space in cochlea and vestibule is clearly visualized (**d**: arrows)

The endolymph fluid, which resides inside the membranous labyrinth, has a composition similar to that of intracellular fluid and is supplied by secretion in the stria vascularis of cochlear duct and vestibular dark cell area. In the inner ear, AQP1-5 was detected in the cochlea, AQP1 and AQP4 were detected in the vestibule, and AQP1-4 and AQP6 were detected in the endolymphatic sac [89]. Meniere’s disease is a disorder of the inner ear that causes vertigo attacks, fluctuating hearing loss, tinnitus, and aural fullness. Meniere’s disease is a complex, heterogeneous disorder in which many underlying factors interact, including anatomical variations in the temporal bone, genetics, autoimmunity, migraine, altered intralabyrinthine fluid dynamics, and cellular and molecular mechanisms. Cytochemical findings include alteration of aquaporin expression, which may cause endolymphatic hydrops, which is a disorder wherein excessive endolymph accumulates in the inner ear and causes damage to the ganglion cells [90, 91]. This endolymphatic hydrops shares the characteristics of dysfunction of dynamics of ISFs in the inner ear, which could be categorized in interstitial fluidopathy. It is interesting that above mentioned COL4A1 related disorder is sometimes accompanied with glaucoma or Meniere’s disease symptoms which can be categorized as interstitial fluidopathies. Endolymphatic hydrops is associated with development of the clinical symptoms, and the hydrops can be visualized with delayed imaging after GBCA administration using heavily T2-weighted FLAIR images. The method visualizes endolymph by subtraction images (hybrid of reversed image of positive endolymph signal and native image of positive perilymph signal (HYDROPS) images) [37, 90, 92] (Fig. 5).

### Idiopathic normal pressure hydrocephalus (iNPH)

In idiopathic normal pressure hydrocephalus (iNPH), intracranial pressure is often maintained in the normal range, although the ventricles are enlarged. It shows characteristic imaging findings such as dilatation of the Sylvian fissure, narrowing of the CSF space in the parietal region, callosal angle, but there are many unclear points regarding the pathophysiology, especially in the kinetics of the CSF and ISF.

A series of studies employing intrathecal administration of GBCA to human subjects for normal pressure hydrocephalus are reported. In the normal pressure hydrocephalus cases, contrast medium was refluxed into the ventricles, which is similar to the known computed tomography cisternography by intrathecal iodine contrast. In addition, in both the normal pressure hydrocephalus cases and the control group, enhancement of the brain parenchyma was observed over time from the enhancement of the CSF space, and the 24-h follow-up showed that the enhancement of the parenchyma tended to be prolonged in the normal pressure hydrocephalus group. These findings have suggested that the hypofunction of the glymphatic system may be related to the pathology of normal pressure hydrocephalus [93]. A study by the Norwegian group demonstrated that GBCA as a CSF tracer distributed centripetally from the surface toward structures in the deep parts of the brain, for which vascular pulsations mediated to CSF seem to play an important role in tracer entry into the brain parenchyma. Additionally, periventricular enhancement due to reflux of tracer into the ventricular system is a typical feature of iNPH. This study also showed that clearance of the GBCA tracer was delayed in the idiopathic normal pressure hydrocephalus cohort [94]. These findings indicate that the idiopathic normal pressure hydrocephalus has altered dynamics of the ISF of the CNS.

A recent pathological study indicated an increased number of pathological mitochondria in astrocytic endfoot processes in patients with iNPH, and the proportion of pathological mitochondria correlated significantly with an increasing degree of astrogliosis and reduced perivascular expression of AQP-4 [95]. A study of normal pressure hydrocephalus cases, which employed the diffusion tensor image analysis along the perivascular space method, evaluated the aforementioned diffusivity limited to the direction of the perivascular space. The report showed that the normal pressure hydrocephalus cases had a lower diffusivity in the direction of the perivascular space than the control cases, suggesting that the ISF/CSF dynamics is impaired in normal pressure hydrocephalus [96].

### Other candidates for CNS interstitial fluidopathies

**Fig. 6:**
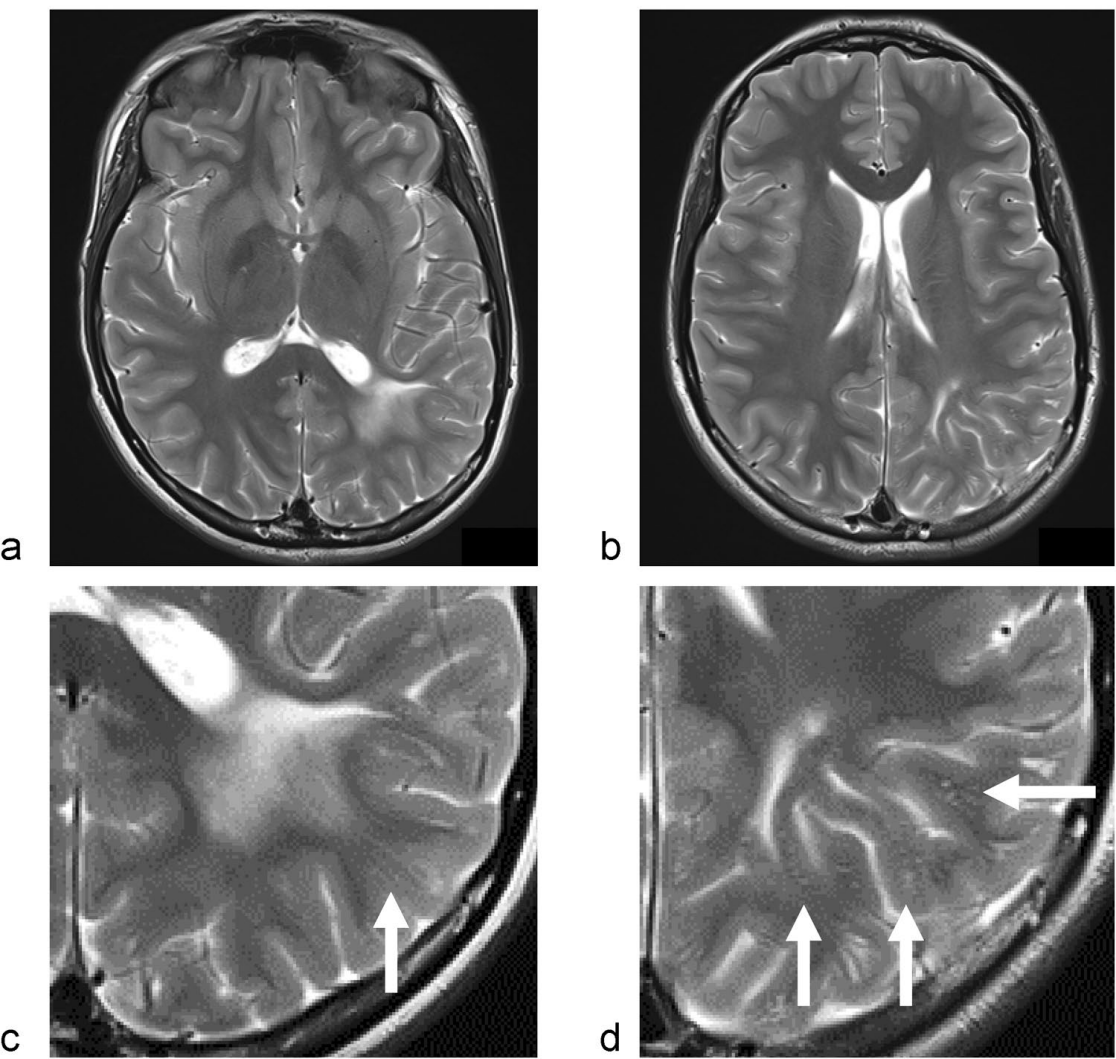
A case with migraine with dilated perivascular space. **a**, **b** T2-WI (axial), **c**, **d** T2-WI (magnified view of **a** and **b**). Fifteen years old female, who has severe headache. T2-WI showed high signal in occipital white matter. There are no other abnormalities on MRA, contrast enhanced MRI, perfusion study, diffusion images or MR spectroscopy, and the case clinically diagnosed as migraine. Note dilated perivascular space on Magnified view (arrows)

Other than the already discussed clinical scenarios, several diseases or disorders are known in which the ISF/CSF dynamics are altered. As shown in the discussion of small vessel disease or TBI, enlarged perivascular space might be one of the markers for the alteration of ISF dynamics. High incidence of enlarged perivascular space are reported found in children with headache, and the enlarged perivascular space is reported to significantly common in children with migraine compared to other causes of headache [97, 98] (Fig. 6). Migraine is reported to have clinical or pathological relationship with the diseases or disorders which were already discussed in this review article as CNS interstitial fluidopathy which has altered dynamics of ISF/CSF including AD [99], sleep disorders [100], ischemic strokes [101], glaucoma [102] and Meniere’s disease [103]. Migraine has an aspect of neuroinflammation, and interstitial space is regarded as one of locations for inflammatory process in migraine [104]. In addition, recent animal experiment revealed the alteration in the ISF dynamics in migraine. An experiment using two-photon microscopy to visualize the perivascular space showed that a single wave of cortical spreading depression, which is a method of animal model for migraine aura, induces a rapid and nearly complete closure of the perivascular space around surface and penetrating cortical arteries and veins lasting several minutes, and gradually recovering over 30 min [105]. On these findings, it is speculated that migraine has aspect of CNS interstitial fluidopathy.

**Fig. 7:**
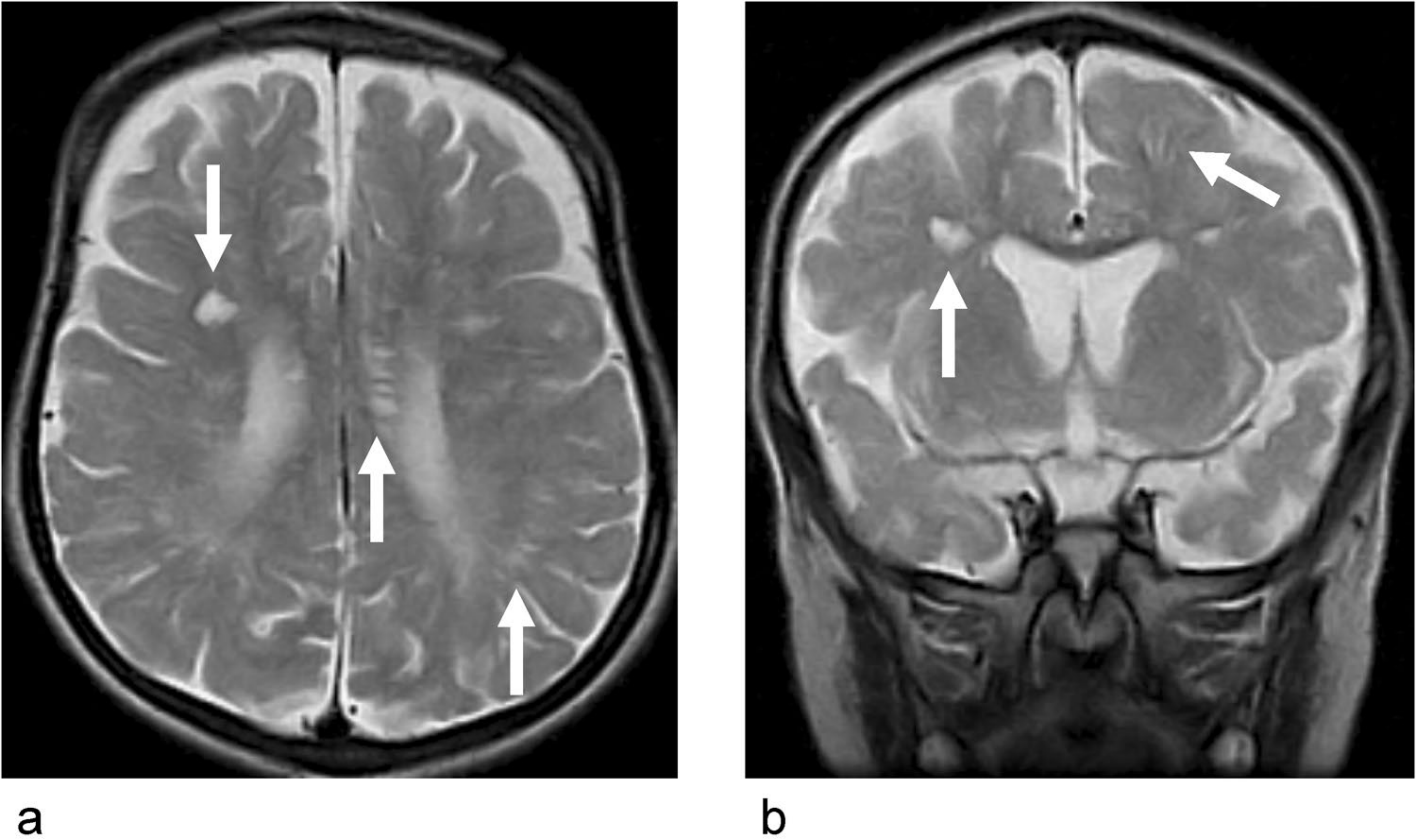
Dilated perivascular space in mucopolysaccharidoses. **a** T2-WI (axial), **b** T2-WI (coronal). Four years old boy, who has contracture of joints, hearing disability and facial dysmorphism. He has been diagnosed as Hunter syndrome by urinary glycosaminoglycans analysis. Axial and coronal T2WI shows periventricular abnormal signal suggesting dilated perivascular space (arrows).

Different types of mucopolysaccharidoses also show a dilated perivascular space (Fig. 7), which is suggestive of pathologies related to the ISFs [106]. Discussions about multiple sclerosis and ISF dynamics are known. In the study of amyloid imaging using PiB PET to detect CSF clearance alterations, clearance in lateral ventricular was significantly lower in patients with multiple sclerosis than that in the healthy controls. The results indicate that there are pathologic changes in ISF and CSF dynamics [47]. Several other disorders which might be related to CSF or ISF dynamics and have not been discussed as a point of interstitial fluidopathy including CSF hypovolemia, leukoaraiosis or periventricular leukomalacia in infants, and investigations are expected for these disorders.

## Conclusion

In this review, we discussed about various diseases or disorders, including sleep disorders, AD/PD, iNPH, traumatic brain injury, glaucoma, and Meniere's disease and other disease or disorders, all of which have never been lumped into a single entity. We, however, believe that they all share a common underlying pathology, namely the “CNS interstitial fluidopathy” or just "fluidopathy" (Table 1). Although these diseases or disorders listed appear as less related, disorders of ISF dynamics is a common characteristic that is impaired dynamics of the fluid in the interstitial space, which is regarded as common space with various functions including mass transport, immune function and intercellular signal transmission. In terms of having the same underlying mechanism, at least partially, they may be mutually helpful in developing treatments and prevention methods [37]. Standing on the pathophysiology discussed in this review, a novel common treatment can be developed for CNS interstitial fluidopathy. For example, cilostazol treatment, which was discussed for AD, are also reported to improve ischemia/reperfusion-induced tight junction disruption in endothelial cells [107] and attenuate ischemia–reperfusion-induced blood–brain barrier dysfunction [108]. Thus, cilostazol would have potential for improving dynamics of the ISF/CSF and could be applied for other diseases or disorders of CNS interstitial fluidopathy. Not just the example above, having a viewpoint of the ISF/CSF dynamics seems to be important for understanding CNS diseases or disorders. We would conclude that it is important to have a viewpoint of the dynamics of the fluid in the interstitial space for understanding CNS diseases or disorders, and it would be possible to develop imaging method or novel common treatment methods for “CNS interstitial fluidopathies.”

**Table 1:** The list of the diseases or disorders which were categorized as “CNS interstitial fluidopathy” in this review article

Sleep disorders
Alzheimer’s disease
Parkinson’s disease
Traumatic brain injury
Stroke
Subarachnoid hemorrhage
Ischemic stroke
Small vessel diseases of CNS
Arteriolosclerosis
Cerebral amyloid angiopathy
Cerebral autosomal dominant arteriopathy with subcortical infarcts and leukoencephalopathy (CADASIL)
COL4A1 mutation related disorders
Glaucoma
Meniere’s disease
Idiopathic normal pressure hydrocephalus
Migraine
Mucopolysaccharidoses
